# Macrophages in the Human Cochlea: Saviors or Predators—A Study Using Super-Resolution Immunohistochemistry

**DOI:** 10.3389/fimmu.2018.00223

**Published:** 2018-02-13

**Authors:** Wei Liu, Matyas Molnar, Carolyn Garnham, Heval Benav, Helge Rask-Andersen

**Affiliations:** ^1^Section of Otolaryngology, Department of Surgical Sciences, Uppsala University Hospital, Uppsala, Sweden; ^2^Immunology, Genetics and Pathology - Biovis Platform, Uppsala University, Uppsala, Sweden; ^3^MED-EL Medical Electronics, Innsbruck, Austria; ^4^R&D, MED-EL GmbH, Innsbruck, Austria; ^5^Head and Neck Surgery, Section of Otolaryngology, Department of Surgical Sciences, Uppsala University Hospital, Uppsala, Sweden

**Keywords:** human, cochlea, macrophages, ionized calcium-binding adaptor molecule 1, structured illumination microscopy, immunohistochemistry

## Abstract

The human inner ear, which is segregated by a blood/labyrinth barrier, contains resident macrophages [CD163, ionized calcium-binding adaptor molecule 1 (IBA1)-, and CD68-positive cells] within the connective tissue, neurons, and supporting cells. In the lateral wall of the cochlea, these cells frequently lie close to blood vessels as perivascular macrophages. Macrophages are also shown to be recruited from blood-borne monocytes to damaged and dying hair cells induced by noise, ototoxic drugs, aging, and diphtheria toxin-induced hair cell degeneration. Precise monitoring may be crucial to avoid self-targeting. Macrophage biology has recently shown that populations of resident tissue macrophages may be fundamentally different from circulating macrophages. We removed uniquely preserved human cochleae during surgery for treating petroclival meningioma compressing the brain stem, after ethical consent. Molecular and cellular characterization using immunofluorescence with antibodies against IBA1, TUJ1, CX3CL1, and type IV collagen, and super-resolution structured illumination microscopy (SR-SIM) were made together with transmission electron microscopy. The super-resolution microscopy disclosed remarkable phenotypic variants of IBA1 cells closely associated with the spiral ganglion cells. Monitoring cells adhered to neurons with “synapse-like” specializations and protrusions. Active macrophages migrated occasionally nearby damaged hair cells. Results suggest that the human auditory nerve is under the surveillance and possible neurotrophic stimulation of a well-developed resident macrophage system. It may be alleviated by the non-myelinated nerve soma partly explaining why, in contrary to most mammals, the human’s auditory nerve is conserved following deafferentiation. It makes cochlear implantation possible, for the advantage of the profoundly deaf. The IBA1 cells may serve additional purposes such as immune modulation, waste disposal, and nerve regeneration. Their role in future stem cell-based therapy needs further exploration.

## Introduction

The inner ear is an enclave protected by solid bone. Despite its proximity to infection-prone areas, it was long thought to lack active immune responses. Instead, the endolymphatic sac, which is an endolymphatic appendage remotely situated, was alleged to dispose of waste material and foreign substances involving immune mechanisms (1–4). The fluid around the apical pole of the sensory cells was previously thought to be cleared *via* a “longitudinal” outlet, thus abating harmful inflammatory responses near the receptors. More recently, immune-reactive cells or tissue macrophages were found in other areas of the inner ear under steady-state conditions (5–8).

It is also ostensible that the human inner ear possesses resident and migratory macrophages [positive for markers CD163, ionized calcium-binding adaptor molecule 1 (IBA1), and CD68] within the connective tissues, neurons, and supporting cells (9). These cells were characterized as macrophage/microglial cells and were assumed to belong to the innate and adaptive immune system (10). “Microglia” may not be the appropriate term for these cells owing to their separate ontogeny, morphology, and expression of surface markers (11). Tissue macrophages seem to be replaced from bone marrow myeloid precursors (6, 7), whereas brain microglia undergo self-renewal during life (12).

Resident macrophages may protect the inner ear *via* surveillance, scavenging, and tissue repair. However, adaptive immune responses may also ensue, which may be potentially hazardous owing to the release of damaging modulators that might result in tissue breakdown and self-destruction. Cochlear macrophages can be recruited from blood-borne monocytes to damaged and dying hair cells induced by noise and ototoxic drugs, aging, and diphtheria toxin-induced selective hair cell degeneration (6, 8, 11, 13–25). Scavengers may reach the sensory epithelium *via* the spiral ganglion (11, 18) or the basilar membrane (BM) (6). These cells may release interferons, inflammatory cytokines, and chemokines *via* the complement cascade. Moreover, supporting cells participate in the disposal of cells, and precise monitoring would seem crucial to avoid self-targeting (26–29).

Cochlear macrophages seem to play important roles in cochlear physiology and pathology. Although their exact roles have not been firmly established, they potentially have both beneficial and detrimental functions. Perivascular-resident macrophage-like melanocytes exist in the stria vascularis (StV) (30, 31) and are seemingly important for maintaining the blood/labyrinth barrier by controlling endothelial tight junctions. Hence, more information is needed about their role in aggravating sensorineural hearing loss (SNHL). How can we avoid triggering their adverse action and exploit their positive effects? Cochlear macrophages may respond adversely in cochlear implantation (CI) and counteract inner ear stem cell engraftment. An unexpected interaction between the innate immune system and cochlear afferents was recently described by Kaur et al. (23). They found that hair cell loss is linked to a chemokine signaling system protecting spiral ganglion neurons. This phenomenon could positively influence neuron rescue following hair cell loss. Whether such coordination prevails in humans remains unknown.

Therefore, we further analyzed human cochlear macrophages using the marker protein IBA1, an actin crosslinking protein in macrophages/microglia associated with membrane ruffling and phagocytosis (20, 32). IBA1 is widely used due to its specificity and expression in both reactive and quiescent microglial cells (33). Directly fixed cochleae were cryosectioned and viewed in a super-resolution structured illumination microscope (SR-SIM). Furthermore, archived semi-thin sections were examined combined with transmission electron microscopy (TEM).

## Materials and Methods

### The Human Cochlea—A Histological Challenge

Table 1 shows patient data and methods of analysis. Studies of the human cochlea are difficult due to the solid bone which requires extensive decalcification. In this study, well-fixed human tissue was obtained with excellent antigen retrieval, allowing the study of protein expression using super-resolution microscopy. An obvious weakness is the limited quantity of tissue and sections.

**Table 1:** Patient data and methods of analysis

Age (years)	PTT	Analysis
40-	50 dB (1–8 kHz)	IF
50-	Normal	IF
70-	50 dB (2–4 kHz)	IF
60-	Normal	IF
40-^a^	Normal	LM, TEM
40-^b^	Normal	LM, TEM

*IF, immunofluorescence; TEM, transmission electron microscopy; PTT, pure tone threshold*.

*^a^Pamulova et al. (40)*.

*^b^Tylstedt and Rask-Andersen (39)*.

In addition, the guinea pig brain was processed for IBA1 staining. The guinea pig brain was dissected out and immersed in fixative. The procedure followed that used for human sections including antibodies and staining.

### Ethic Statements

The study of human materials was approved by the local ethics committee (no. 99398, 22/9 1999, cont, 2003, no. C254/4; no. C45/7 2007, Dnr. 2013/190), and patient consent was obtained. The study adhered to the rules of the Declaration of Helsinki. Archival sections from adult cochleae were used (34, 35). Guinea pig cochleae and brain were analyzed in parallel as controls. Ethical consent was obtained from the local ethical committee of Uppsala for animal use. The guinea pig study’s protocol was approved by the Regional Animal Review Board of Uppsala, Sweden and guinea pig C98/12 and C66/16.

### Fixing and Sectioning of Human Cochleae for Immunohistochemistry

In this study, we used archival human material used in earlier publications (34, 35). Four cochleae (two from males and two from females; aged 44–72 years) were dissected as a whole piece instead of being discarded during petroclival meningioma surgery (two with normal pure tone thresholds for their age and two with moderate SNHL due to a life-threatening tumor compressing their brain stem). In the operating room, the cochleae were immediately placed in 4% paraformaldehyde and diluted in 0.1 M phosphate-buffered saline (pH 7.4). After a 24-h fixation, the fixative was replaced with PBS and then with a 0.1 M Na-ethylene-diamine-tetra-acetic acid solution at pH 7.2 for decalcification. After 4 weeks, the decalcified cochleae were rinsed with PBS. The cochleae were embedded in Tissue-Tek OCT (Polysciences), rapidly frozen, and sectioned at 8–10 μm using a Leica cryostat microtome to obtain frozen sections. The frozen sections were collected on gelatin/chrome alum-coated slides and stored below −70°C before immunohistochemistry.

### Antibodies and Immunohistochemistry

Table 2 shows the series of antibodies used in this study. The antibody against type IV collagen was used to demarcate the basal lamina (BL) of neurons, blood vessels, and epithelium. We used a polyclonal antibody (1:25, goat ab, AB769, Millipore). For residents’ macrophages, we used antibody against IBA1 (polyclonal, 1:100, rabbit, PA527436 from Invitrogen). Specificity was proven by IBA1 antibody blotting (36). Fractalkine antibody was a monoclonal antibody (1:100, mouse, MAB3651, R&D Systems). This antibody specificity antibody was verified in western blotting experiment (37). Information about the other primary and secondary antibodies is shown in Table 2.

**Table 2:** Antibodies used in the study.

Primary antibody	Type	Dilution	Host	Catalog number	Producer
Collagen IV	Polyclonal	1:25	Goat	AB769	Millipore
Laminin β2	Monoclonal	1:100	Rat	#05-206	Millipore
Ionized calcium-binding adaptor molecule 1	Polyclonal	1:100	Rabbit	PA527436	Invitrogen
Tuj 1	Polyclonal	1:200	Rabbit	#04-1049	Millipore
Tuj 1	Monoclonal	1:200	Mouse	MAB1637	Millipore
CX3CL1	Monoclonal	1:100	Mouse	MAB3651	R&D Systems
CX3CR1	Polyclonal	1:50	Rabbit	#PA5-19935	Invitrogen
P2Y12	Polyclonal	1:50	Rabbit	#PA5-34079	Invitrogen
Major histocompatibility complex class II	Monoclonal	1:50	Mouse	#MA5-11966	Invitrogen

**Secondary antibody**		**Dilution**	**Host**	**Catalog number**	**Producer**
Anti-Mouse IgG H&L		1:400	Goat	A21422	Invitrogen
Alexa Fluor^®^ 555					
Anti-Rabbit IgG H&L		1:400	Goat	A11008	Invitrogen
Alexa Fluor^®^ 488					
Anti-Goat IgG H&L		1:400	Donkey	Cat # A-21432	Invitrogen
Alexa Fluor^®^ 488					
Anti-Mouse IgG H&L		1:400	Donkey	Cat # A-21202	Invitrogen
Alexa Fluor^®^ 488					
Anti-Rabbit IgG H&L		1:400	Donkey	Cat # A-31572	Invitrogen
Alexa Fluor^®^ 555					

### Imaging and Photography

The stained sections were first investigated with an inverted fluorescence microscope (Nikon TE2000) equipped with a spot digital camera with three filters (for emission spectra maxima at 358, 461, and 555 nm). Image-processing software (NIS Element BR-3.2, Nikon), including image merging and a fluorescence intensity analyzer, was installed on a computer system connected to the microscope. For laser confocal microscopy, we used the same microscope equipped with a three-channel laser emission system. The optical scanning and image-processing tasks were performed using Nikon EZ-C1 (ver. 3.80) software and included the reconstruction of *Z*-stack images into projections and 3-D images. Super-resolution structured illumination microscopy (SR-SIM) was performed using a Zeiss Elyra S.1 SIM system and a 63×/1.4 oil Plan-Apochromat objective (Zeiss), sCMOS camera (PCO Edge), and ZEN 2012 software (Zeiss). Multicolor SR-SIM imaging was achieved with the following laser and filter setup: first channel—405 nm laser excitation and BP 420-480 + LP 750 filter; second channel—488 nm laser excitation and BP 495-550 + LP750 filter; third channel—561 nm laser excitation and BP 570-620 + LP 750 filter. To maximize the image quality, five grid rotations and five phases were used for each image plane and channel. The grid size was automatically adjusted by the ZEN software for each wavelength of excitation. SR-SIM images were processed with ZEN software using automatic settings and theoretical point spread function (PSF) calculations. From the SR-SIM dataset, 3-D reconstruction was performed using the Imaris 8.2 (Bitplane, Zürich, Switzerland). A bright-field channel was merged with fluorescence to visualize the cell borders. The microscope is capable of achieving a lateral (*X*–*Y*) resolution of ≈100 nm and an axial (*Z*) resolution of ≈300 nm (38). The resolution of the SIM system in BioVis (Uppsala) was measured with sub-resolution fluorescent beads (40 nm, Zeiss) in the green channel (BP 495-550 + LP750). An average PSF value was obtained from multiple beads with the built-in experiment PSF algorithm of the ZEN software. The typical resolution of the system was 107 nm in the *X*–*Y* plane and 394 nm in the *Z* plane. 3-D reconstructions of collagen IV and IBA1 protein expression were made. Both signals were reconstructed with surface rendering mode using the Imaris 8.2 software.

### Transmission Electron Microscopy

Archival sections were re-analyzed from specimens collected during surgery and earlier publication (39). In the apical part, where the ganglion cells accumulate, ultrathin serial sections with a thickness of about 800 Å were made and examined by TEM. Three hundred sixty-five consecutive serial thin sections were initially stained with uranyl acetate and lead citrate and then observed in a JEOL 100 SX transmission electron microscope. The specimens had been fixed in 3% phosphate-buffered glutaraldehyde, pH 7.4, and rinsed in 0.1 M cacodylate buffer, followed by fixing with 1% osmium tetroxide at 4°C for 4 h. The specimens were infiltrated with Epon resin in a vacuum chamber for 4 h. One cochlea was dissected out for the study of the innervation pattern of the apical turn of the human cochlea (40). The cochlea was placed in 3% sodium phosphate-buffered glutaraldehyde for 48 h. Thereafter, it was placed in 0.1 mol/L sodium ethylene-diamine tetra-acetic acid for 4 weeks at room temperature. Decalcification was checked by radiography. The tissue was rinsed and stained with 1% sodium phosphate-buffered osmium tetroxide for 1 h. The apical turn of the cochlea was cut away with small scissors through the modiolus. The tissue was dehydrated and embedded in Epon (Resolution Performance Products, Houston, TX, USA). Semi-thin sections were cut with a glass knife perpendicular to the long axis of the modiolus from the level of the spiral ganglion to the apical top of the organ of corti (OC) and the basilar lamina. Every 10th section was taken up and placed on a paraffin-coated film for re-embedding for TEM.

## Results

### Semi-Thin Sections of OC

**Figure 1:**
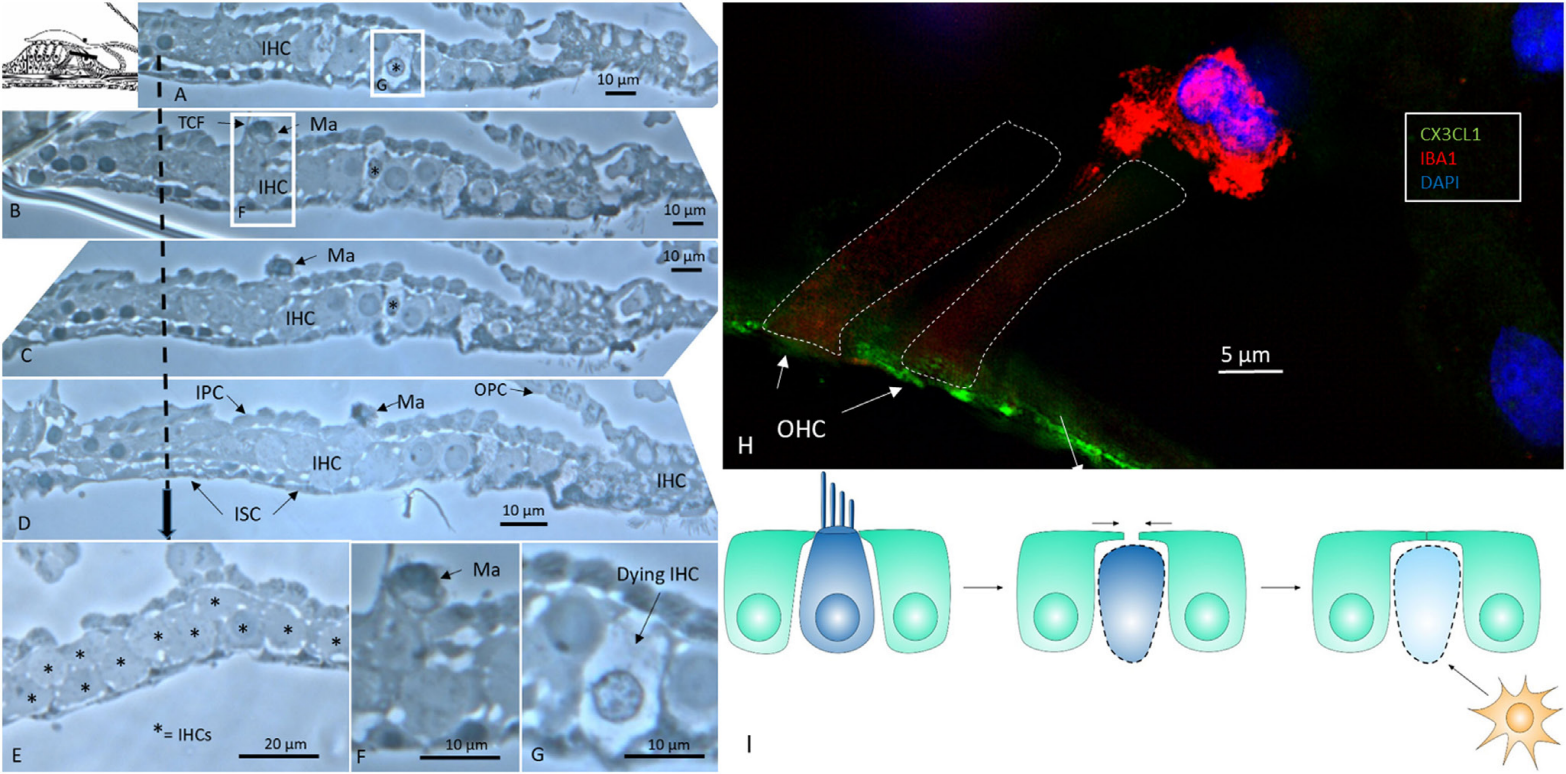
Human organ of corti (OC) contains migrating macrophages. **(A–D)** Horizontal serial sections (1 μm) of the apical turn of the OC dissected from a middle-aged female with normal hearing in both ears during surgery for petroclival meningioma (40). **(B,F)** Dendritic macrophage (Ma) with lysosomes can be seen in the corti tunnel near a degenerating inner hair cell (IHC). **(E)** Many IHCs (supernumerary) are present beneath the reticular lamina. **(F)** Framed area in panel **(B)** is shown in higher magnification in panel **(F)**. **(G)** Framed area in panel **(A)** is viewed at higher magnification in panel **(G)** and displays an IHC undergoing degeneration. TCF, tunnel crossing fiber; OPC, outer pillar cell; IPS, inner pillar cell; ISC, inner sulcus cell; IPhC, inner phalangeal cell (1% osmium tetroxide and toluidine blue staining). The broken arrow displays top-down sectioning. **(H)** Immunofluorescence of an ionized calcium-binding adaptor molecule 1 (IBA1)-IR cell associated with an outer hair cell in the OC. The reticular lamina expresses fractalkine. **(I)** Hypothetical representation of macrophage disposal of dying hair cells in the human OC. Hair cells retract beneath the reticular lamina and are removed by activated macrophages.

An examination of horizontal serial semi-thin sections from archival specimens (individual with normal PTA) showed few active dendritic, lysosome-containing macrophages in the OC. The macrophages found resided in the corti tunnel, adhering to the lateral cell surface of the inner pillars near a few dying inner hair cells (IHCs) (Figure 1). The pillars formed a tight cell boundary that was at times discontinuous. The origin of the dendritic macrophages could not be determined. In the apical regions that were viewed in series, crowded supernumerary IHCs were seen (Figure 1E). These cells were bordered by inner sulcus and inner phalangeal cells. Several IHCs were separated by thin cytoplasmic ramifications from the supporting cells, whereas others were juxtaposed, lacking a separate barrier. One cell lay in direct contact with the tunnel crossing fibers (Figures 1B,F). The preservation of cells was good, and the few degenerating IHCs were therefore not considered to represent postmortal degeneration or autolysis.

### Confocal and Super-Resolution Structured Illumination Microscopy (CF, SR-SIM)

**Figure 2:**
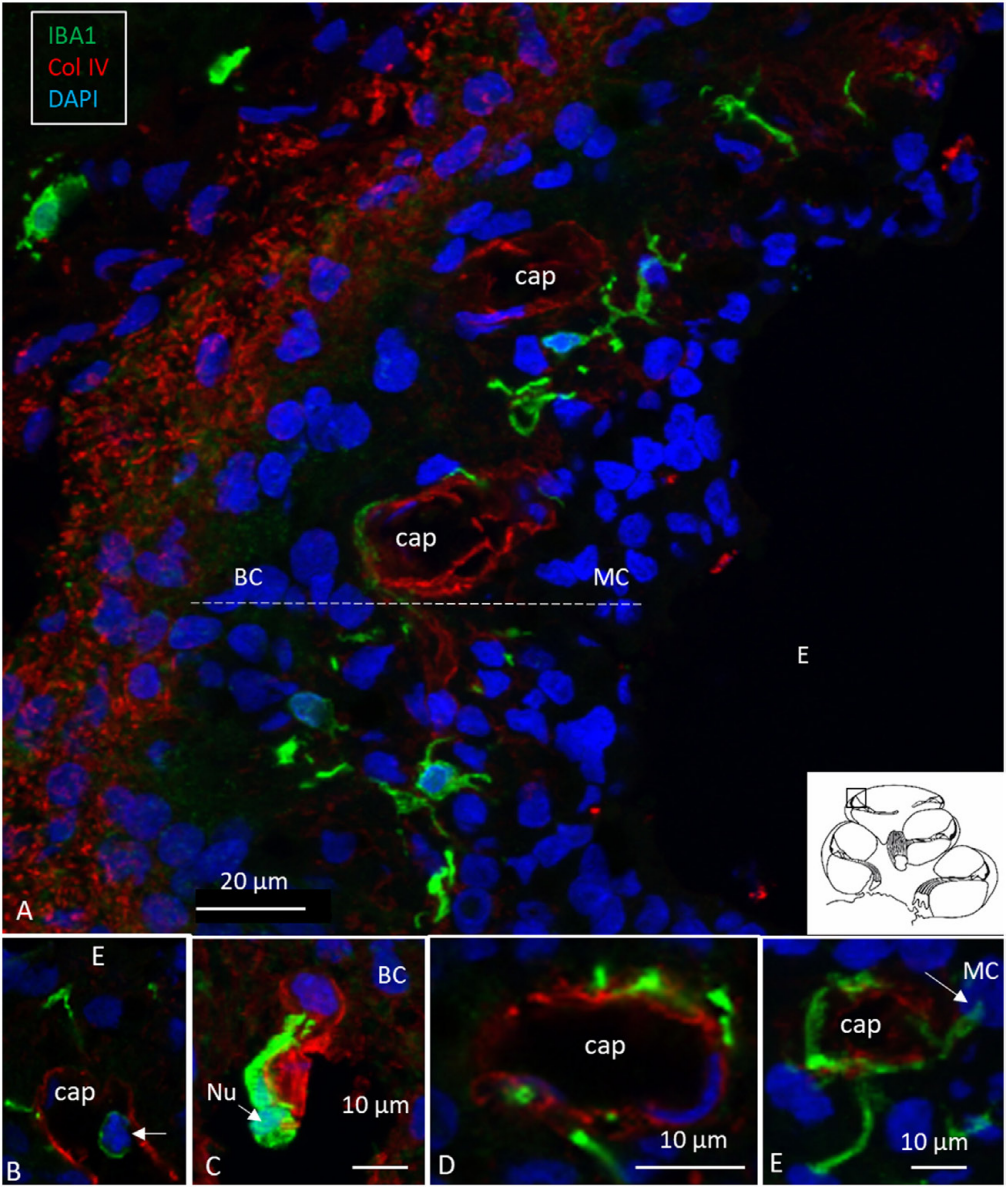
Human stria vascularis contains a myriad of macrophages closely related to blood vessels. **(A)** Immunofluorescence of ionized calcium-binding adaptor molecule 1 (IBA1)-IR cells in the lateral wall of the apical turn of the human cochlea. Most cells are located in the stria vascularis (broken line), but there are also cells in the spiral ligament. **(B–E)** There are many perivascular cells. There is one intra-capillary IBA1 cell **(B)**. Col. IV, collagen IV; Cap, capillary; E, endolymph; MC, marginal cells; BC, basal cell; Nu, nucleus.

**Figure 3:**
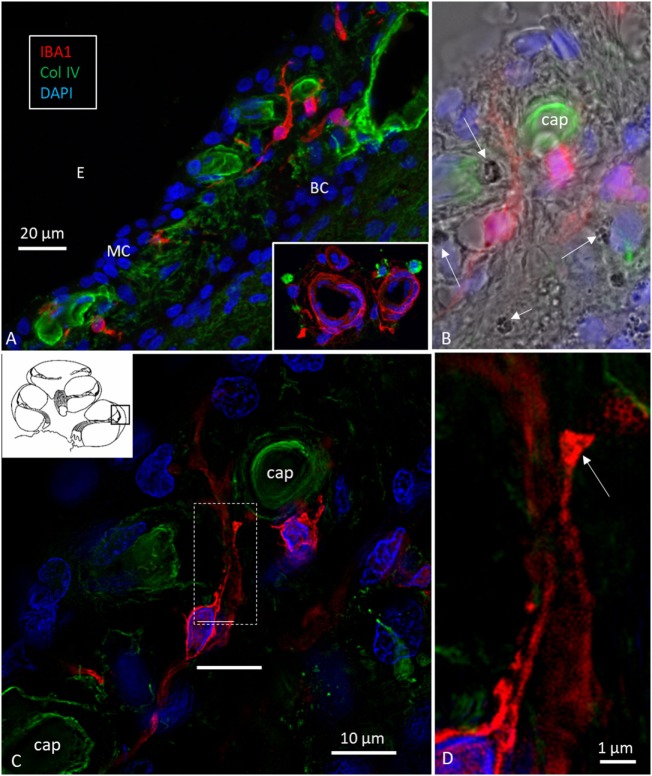
Ionized calcium-binding adaptor molecule 1 (IBA1) cells in the stria vascularis are not intermediate cells (ICs). Confocal microscopy **(A,B)** and SR-SIM [**(C,D)** maximal intensity projections] images of IBA1-positive cells in the lateral wall of the human cochlea. Their long pseudopodia reach the wall of the strial capillaries and MCs. A terminal bulb can be seen at the capillary wall [arrow in panel **(D)**]. The IBA1 cells contain no melanin granules [arrows in panel **(B)**], suggesting they are not ICs. MC, marginal cells; BCs, basal cells; E, endolymph; Cap, capillary of the StV. Inset in panel **(A)** shows perivascular macrophages (IBA1, green) in the wall of the scala tympani.

Ionized calcium-binding adaptor molecule 1-expressing cells were located in the lateral wall, including the spiral ligament, scala vestibule (SV) and scala tympani (ST), spiral limbus, endosteum, tympanic covering layer (TCL), spiral lamina, spiral and vestibular ganglion (VG), dendrites, and central axons (Table 3). In three specimens, an IBA1-positive cell was found in the OC. A few IBA1 cells were found in the OC and one such cell was found to be closely associated with the base of an outer hair cell (Figure 1I). Staining was intense and specific. Their morphology varied greatly from being multi-branched to round or elongated depending on their location and their nuclear chromatin typically expressed IBA1. Cell processes that contacted adjacent vessels generally showed a terminal bulb or podosome. In the stria vascularis (StV), they were mostly located in the mid-zone near and around the blood vessels and intermediate cells (ICs) (Figures 2, 3). An IBA1-positive intraluminal cell was observed (Figure 2B). Macrophages were generally unrelated to melanin granules (Figure 3B). The cells were endowed with dendritic processes that could extend even between marginal cells, almost reaching the endolymph. No IBA1 cells were found in Reissner’s or tectorial membranes. A few cells were located in the spiral ligament, often around blood vessels. No IBA1 cells were located among the type I fibrocytes. Several were located among type II, III, and IV fibrocytes. Few cells were located among the type V fibrocytes. The wall of the SV and ST contained IBA1 cells. Notably, there was perivascular distribution of cells around vessels in the lower aspect of the ST (Figure 3A, inset).

**Table 3:** Distribution of ionized calcium-binding adaptor molecule 1 (IBA1)-IR cells in the human cochlea.

Stria V	Spiral lig. III	Spiral lig. II, IV, V	SG	Spiral limb.	TM/RM	Bone		
+++	++	+	+++	+	−	+		

**Oss. lam**.	**Wall ST**	**Wall SV**	**TCL**	**OC**	**Axon**	**Dendrite**	**VG**	**VN**

++	++	++	++	+	+++	+++	+++	+++

*Stria V., stria vascularis, IBA1 cells are mostly associated with blood vessels; Spiral lig., spiral ligament, the fibrocytes are classified into I–V types; SG, spiral ganglion; VG, vestibular ganglion; Spiral limb., spiral limbus; RM, Reissner’s membrane; TM, tectorial membrane. Bone, endosteal cochlear bone moderately stained with green fluorescence and overlap the red fluorescent labeled collagen IV and lack branched feature of macrophages making it hard to distinguish from vascular endothelial and smooth muscle cells; Oss. Lam., osseous spiral lamina; wall ST, wall of scala tympani, thin layer with Iba1 cell close to the perilymph; wall SV, wall of scala vestibuli, Iba1 cells are mainly located near the bone; TCL, tympanic covering layer; OC, organ of corti; axon and dendrite; not including nerve beyond the fundus in the internal acoustic meatus and most peripheral part of the afferent dendrites that are in the table belongs to the osseous spiral lamina (Oss. Lam.)*.

*A similar pattern of distribution and consistent morphology of IBA1 cells were found in the immunohistochemically analyzed specimens from all four cochleae*.

### IBA1 Cells in the Human Spiral Ganglion

**Figure 4:**
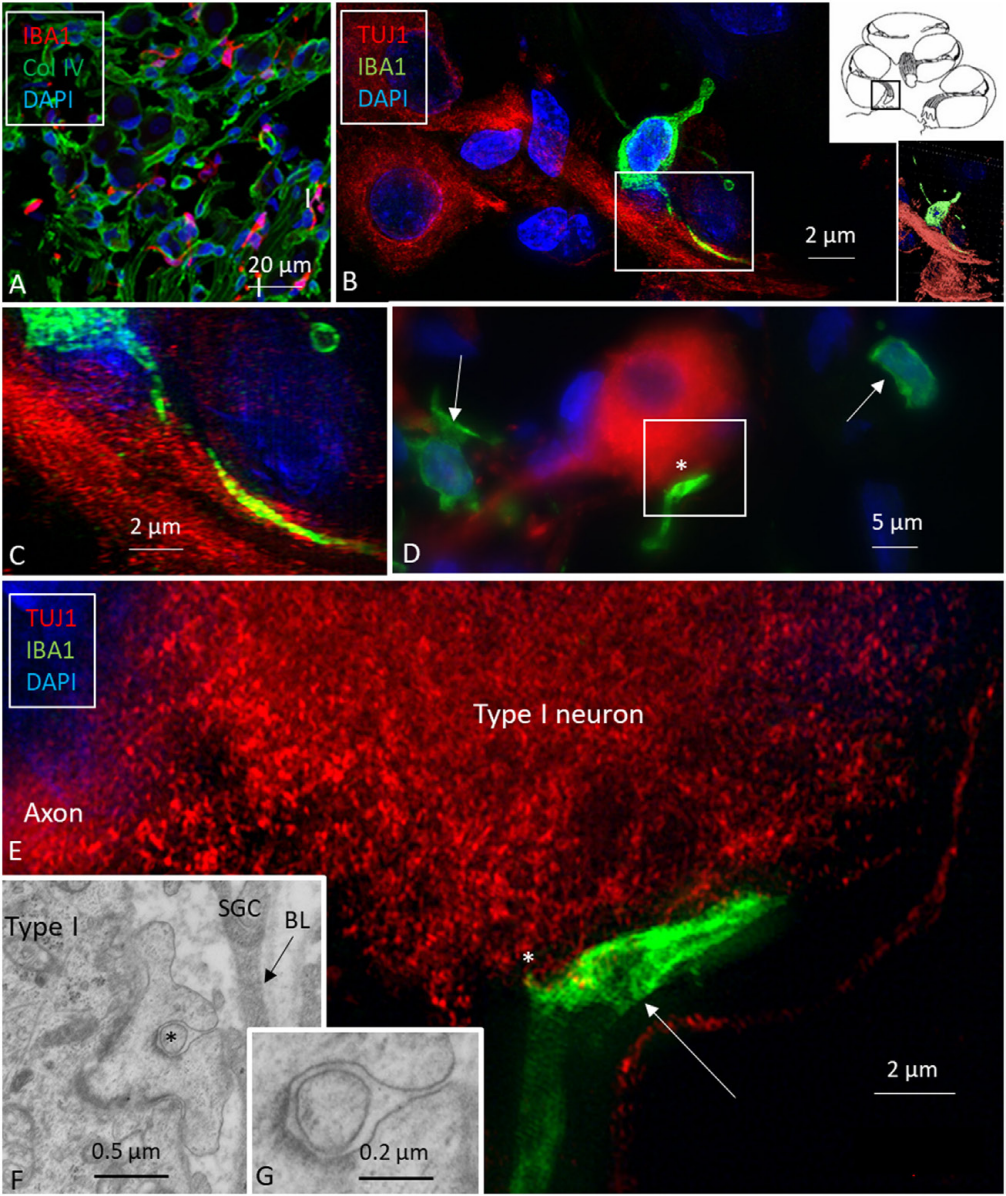
Ionized calcium-binding adaptor molecule 1 (IBA1) macrophages are physically related to spiral ganglion cells. **(A)** Confocal microscopy of immunohistochemistry for collagen IV and IBA1 in the spiral ganglion of the basal turn of the human cochlea. **(B)** SR-SIM of an IBA1-immunoreactive cell that projects into the cytoplasm of an axon process. Framed area is shown in higher magnification in panel **(C)**. Inset shows 3D video reconstruction shown in Video S1 in Supplementary Material. **(D)** Unprocessed SR-SIM showing TUJ1-positive type I spiral ganglion cell with associated IBA1 cell process (*) shown in panel **(E)**. Macrophage cell nuclei typically expressed IBA1 protein. **(E)** SR-SIM (maximal intensity projection) of the synapse-like IBA1 process facing the surface of the type I ganglion cell. A thin cell process (arrow) can be seen projecting into the neuron. **(F, G)** Transmission electron microscopy of unidentified cell processes directly facing the type I ganglion cell. There are cell membrane specializations and signs of possible cell fusion.

**Figure 5:**
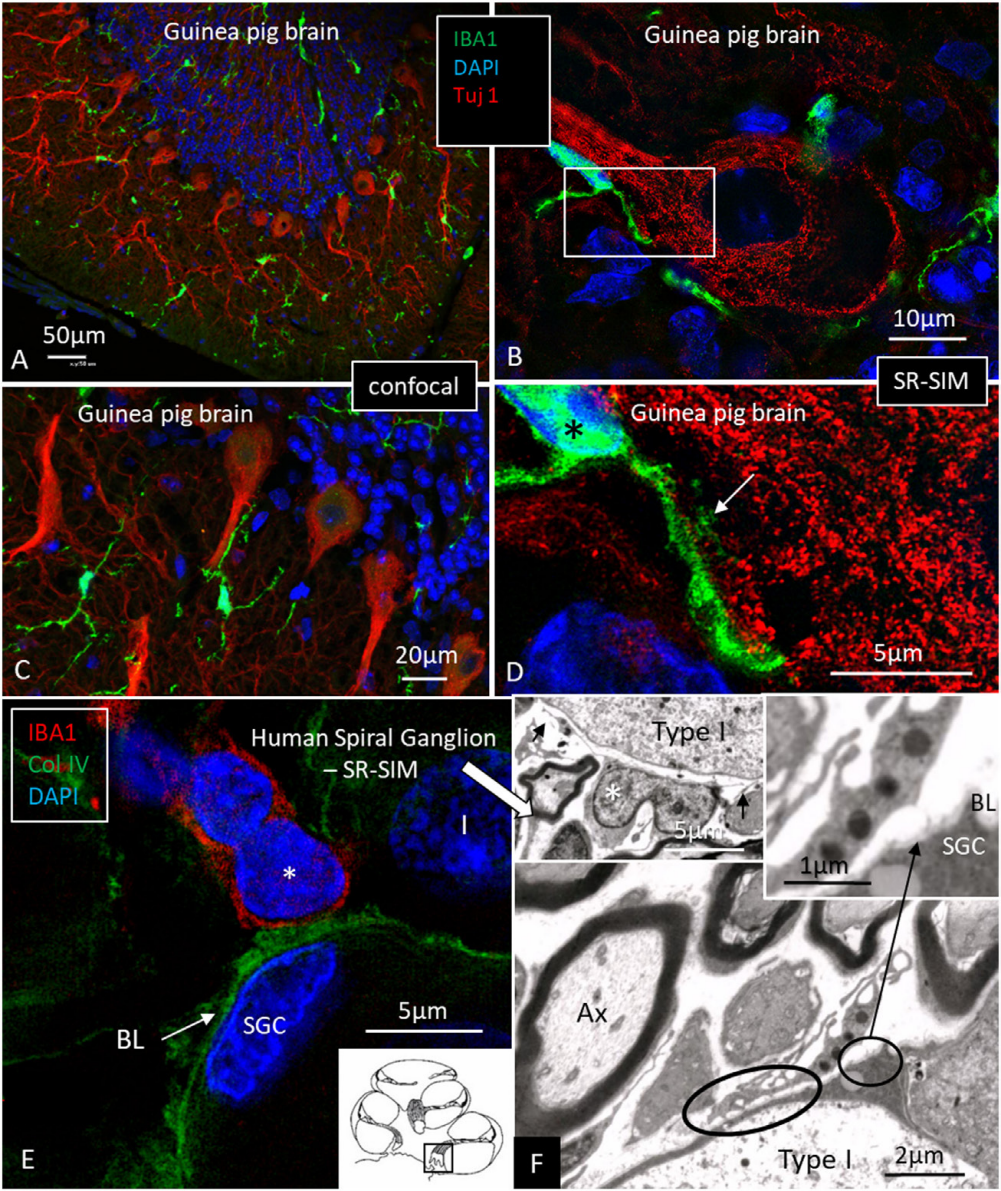
Ionized calcium-binding adaptor molecule 1 (IBA1) macrophage interaction in the brain and ear. **(A,C)** Confocal microscopy of IBA1 and TUJ1 immunoreactivity in the guinea pig bran. IBA1 cells are seen both in the white and gray matter. **(B)** SR-SIM (maximum intensity projection) show IBA1 cells closely related to the surface of the TUJ1-positive neurons. Framed area is magnified in panel **(D)**. **(D)** Higher magnification showing guinea pig brain neurons. IBA1-positive cells are seen in close association with the dendritic processes (arrow). The IBA1 cell nucleus express IBA1 protein (*). **(E)** SR-SIM of the human spiral ganglion showing IBA1-immunoreactive cell (*) physically related to the collagen IV basal lamina (BL) surrounding the satellite glial cell (SGC). **(F)** Transmission electron microscopy of human spiral ganglion shows cells believed to represent macrophages (*). They show slender processes (arrows) and contain typical electron-dense bodies (left inset). Cytoplasmic processes project against the outer surface of the SGCs (arrow in right inset). Type I, type I spiral ganglion cell soma; Ax, axon.

**Figure 6:**
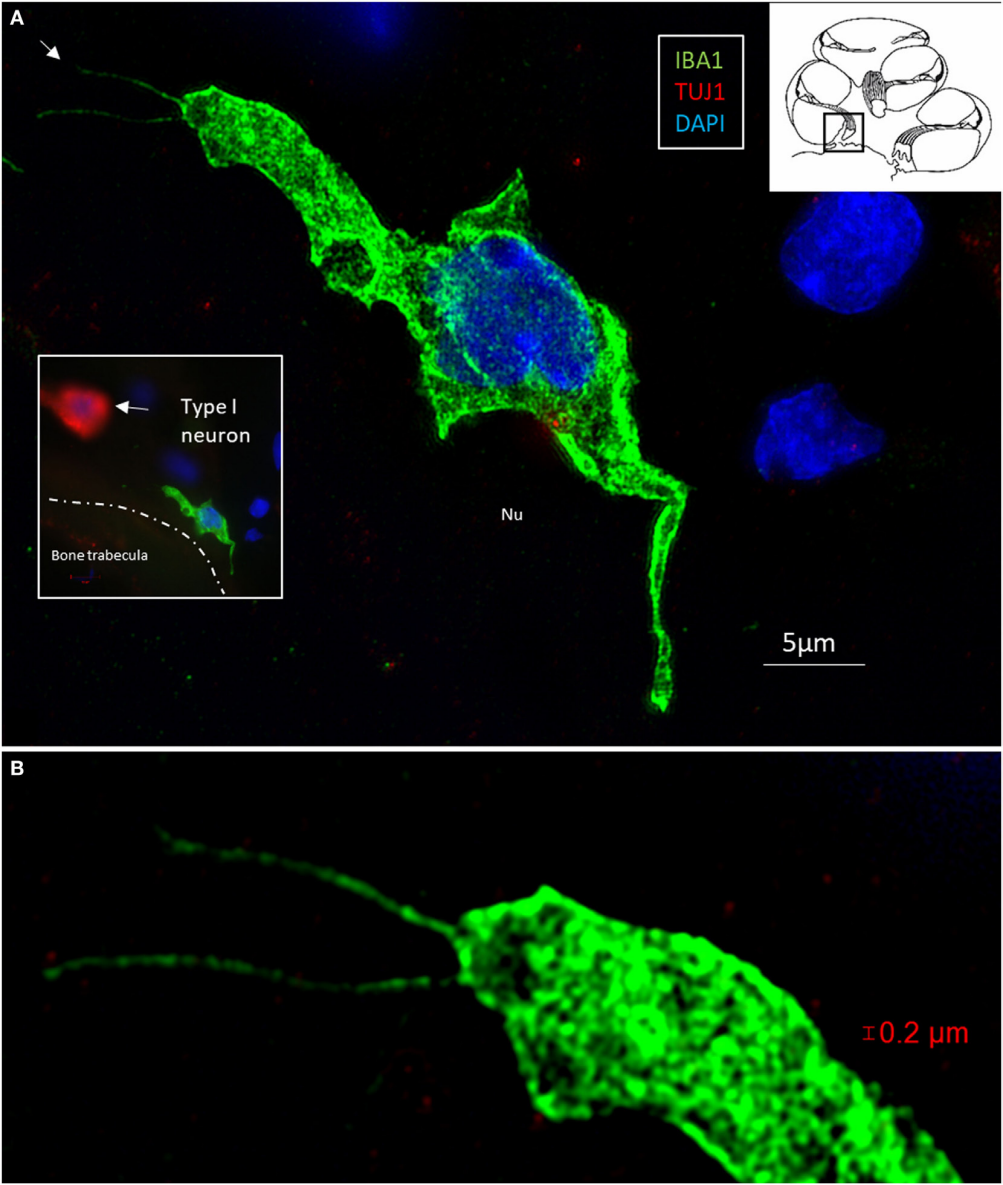
**(A)** Surveilling macrophages with projecting “antennae” exist around the human type I cells. **(A)** SR-SIM (maximum intensity projection) of an ionized calcium-binding adaptor molecule 1 (IBA1)-positive macrophage at the bone trabecula in Rosenthal canal of the first turn of the cochlea. Inset shows relationship to a TUJ1-positive neuron. **(B)** Higher magnification shows “horn”-like projections (arrow). Cytoplasmic vesicles as well as its nucleus contain IBA1 protein.

Multiple IBA1 cells were found in the spiral ganglion, often associated with the satellite cells of the type I cells (Figure 4). The macrophages had pseudopodia and the cell nuclei were often folded and expressed IBA1. Macrophages were also directly associated with axons and dendrites within Rosenthal’s canal (Figures 4B,C) as well as the outer membrane of the spiral ganglion cells (Figures 4D,E). The macrophages frequently adhered to the BL surrounding the satellite cells. Several were situated at the axonal and dendrite entry zones. Synapse-like endings were seen facing TUJ1-positive nerve soma suggesting a location directly on the cell membrane (Figure 4E). A similar association was seen in brain neurons between adhering microglia and the dendrite entry zone (Figures 5A–D). In the human spiral ganglion, cell projections located between the satellite glial cell layer and nerve soma could be observed at TEM (Figures 4F,G). These studies now revealed the particular morphology of the macrophages. Their large number, characteristic nuclei, long processes, and close relationship with neurons made it now possible to now identify these cells at the ultrastructural level (Figure 5). In addition, these cells displayed typical electron-dense vesicles possibly representing lysosomes (Figure 5F). Remarkable phenotypic variants of IBA1 cells were found in the Rosenthal’s canal using SIM. These cells were obviously migrating and independent of the spiral ganglion cells. They displayed intracytoplasmic vesicles and thin (0.2 μm) “antenna”-like processes (Figure 6).

### Macrophages in Central and Peripheral Axons

**Figure 7:**
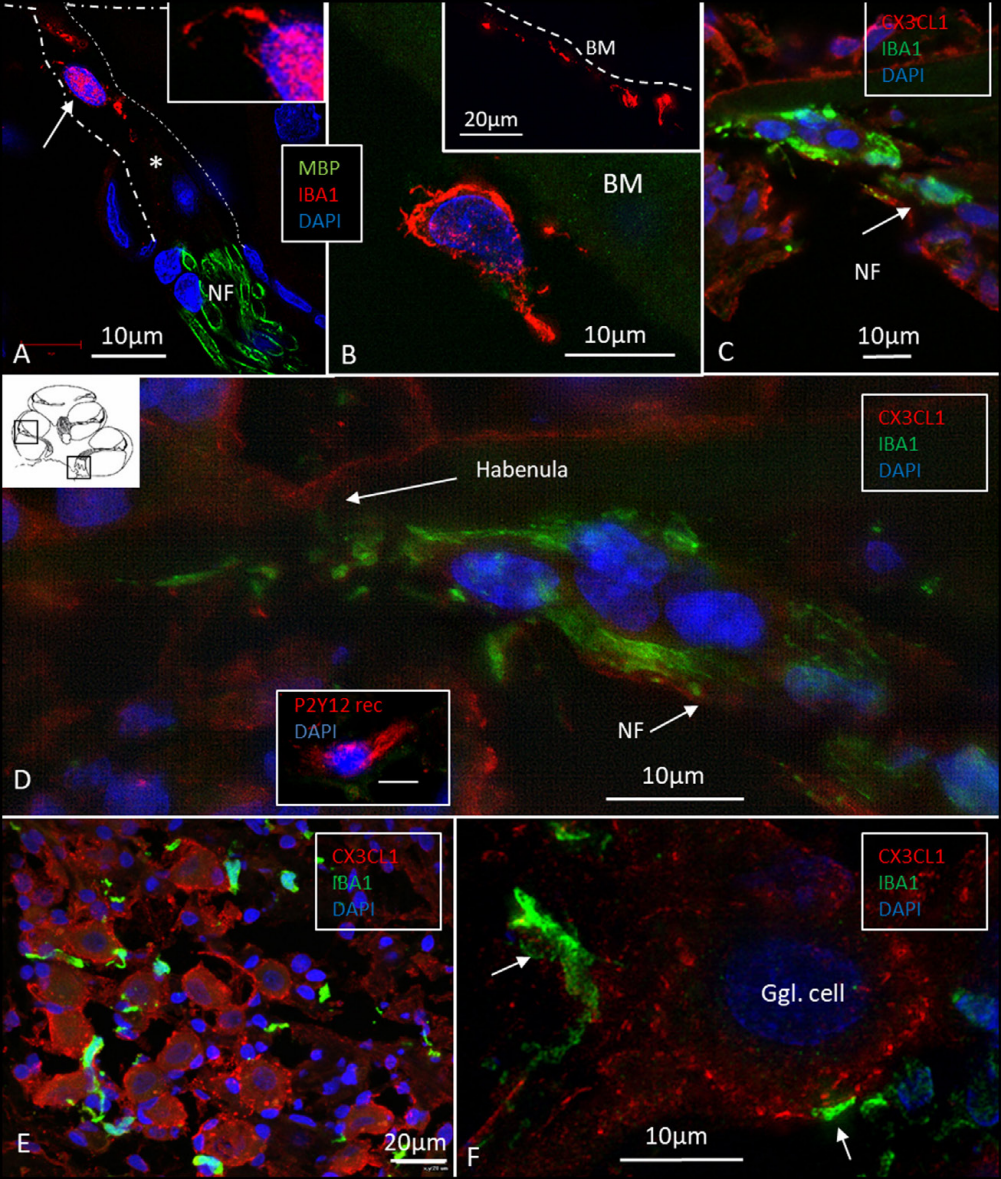
Ionized calcium-binding adaptor molecule 1 (IBA1) cells accumulate at habenula perforate and fractalkine is expressed in the spiral ganglion. **(A)** SR-SIM of IBA1 and myelin basic protein (MBP) expression at the habenula perforata of the human cochlea. A macrophage is shown around the unmyelinated fibers. It has a thin projection against the habenular opening (inset). **(B)** The tympanic covering layer also contains IBA1-positive cells firmly attached to the inferior surface of the basilar membrane (BM). **(C)** Confocal microscopy of numerous IBA1-positive cells around unmyelinated portion of the acoustic nerve bundle (arrow). There is no strong fractalkine expression in the organ of corti. **(D)** Inset in panel **(D)** shows a cell positive for the P2 Y12 receptor. SR-SIM (maximal intensity projection) at corresponding location showing IBA1 cells with thin projection into the habenular opening. **(E)** Confocal microscopy of IBA1 and fractalkine expression in the human spiral ganglion (basal turn). **(F)** SR-SIM (maximum intensity projection) of fractalkine expression with IBA1-positive macrophages surrounding the type I ganglion cell.

**Figure 8:**
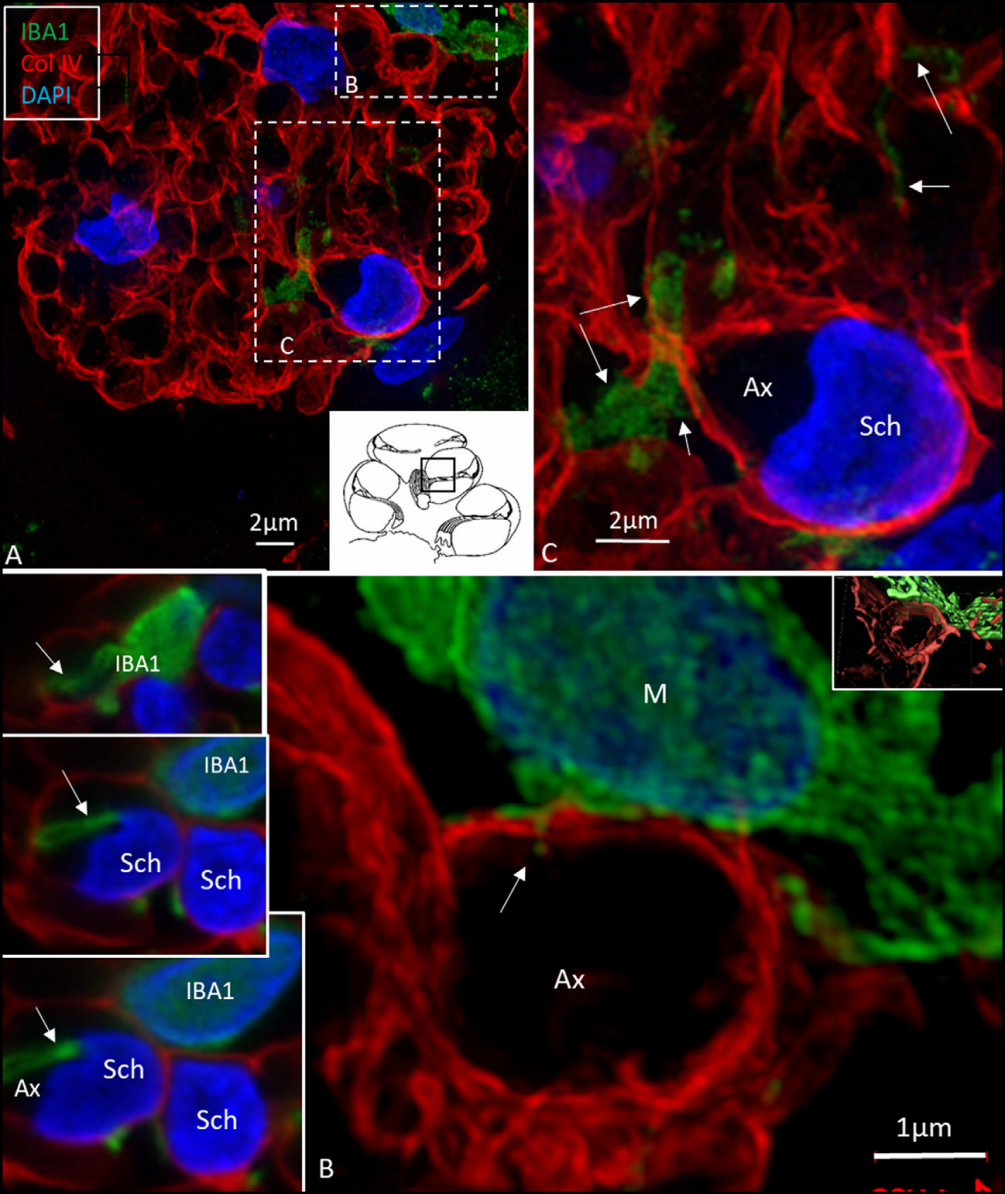
Ionized calcium-binding adaptor molecule 1 (IBA1) cells perforate basal lamina (BL) of Schwann cells. **(A)** SR-SIM (maximum intensity projection) of IBA1 macrophages (M) in a cross-sectioned nerve bundle in the spiral lamina (second turn, inset). Framed areas are shown in higher magnification in panels **(B,C)**. Macrophages (M, green) send projections to adjacent axons (Ax). **(B)** An IBA1 cell process penetrates the BL of a Schwann cell (arrow). Inset shows 3D video reconstruction in Video S2 in Supplementary Material. Serial optical sections **(B)** show IBA1 cell process entering the peri-neuronal space (insets, arrows). **(C)** IBA1 cells send many projections to surrounding axons in the spiral lamina (arrows). Sch, Schwann cell.

**Figure 9:**
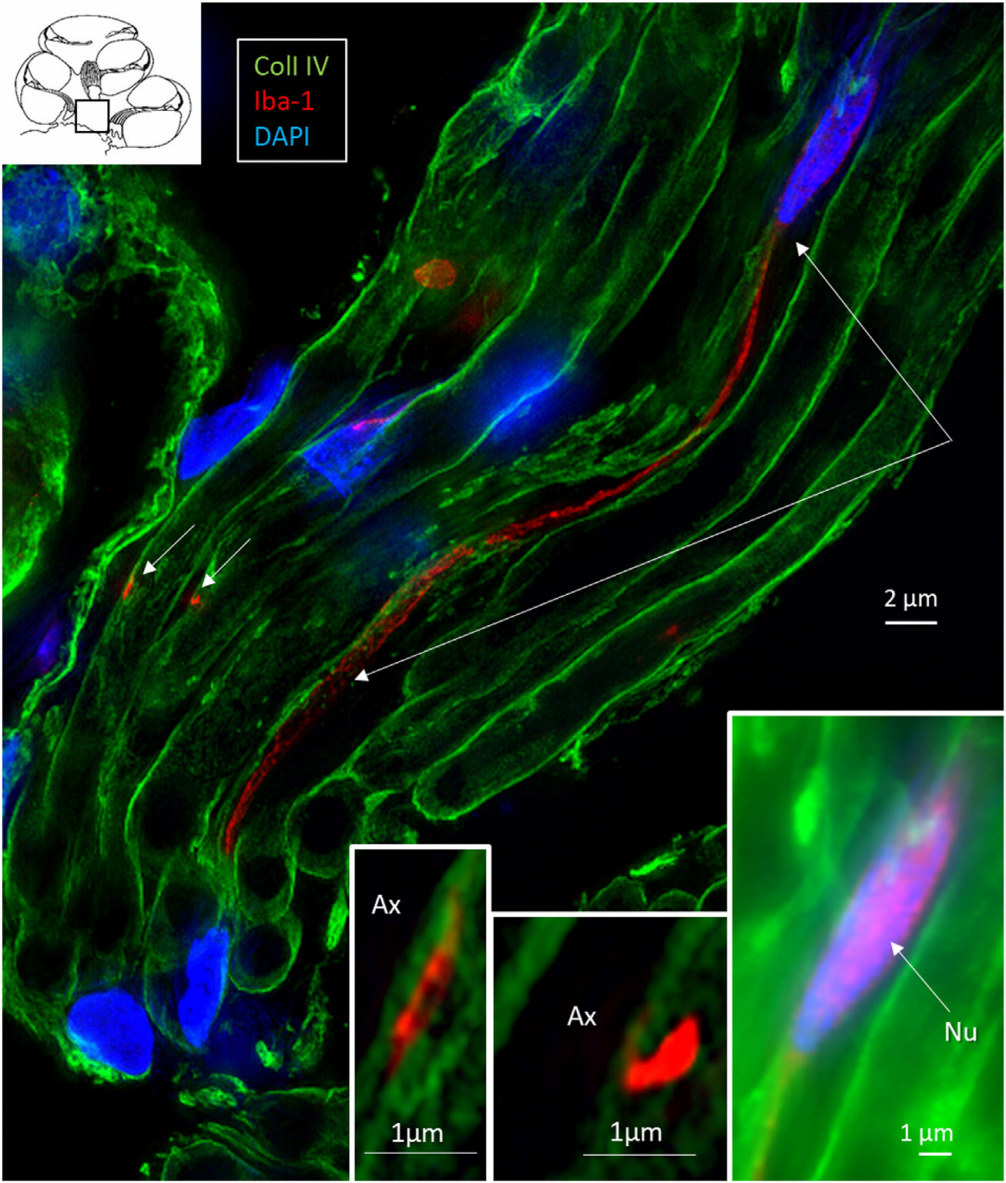
Central auditory nerve axons are connected to ionized calcium-binding adaptor molecule 1 (IBA1)-positive processes. SR-SIM (single optical section) shows IBA1-positive macrophages among central axons in the cochlea (inset, framed area). The IBA1 protein is also expressed in the nucleus (inset right). These macrophages project thin branches that contact adjacent axons (short arrows, magnified in left and middle insets). Nu, nucleus.

Elongated IBA1 cells were also associated with central axons within the modiolus and peripheral dendrites in the osseous spiral lamina (Figures 7–9). IBA1 cells along the central axons were “worm”-like and measured up to 50 μm with a diameter of approximately 0.5 μm (Figure 9). Their nuclei were oblong and expressed IBA1. They displayed thin processes reaching several neighboring fibers, of which several had a terminal cone.

In the osseous spiral lamina, collagen IV and IBA1 staining showed that macrophage pseudopodia could extend through the BL of the Schwann cells, but the relationship to the myelin layer was more uncertain. Thus, the IBA1 cells seemed to be in physical contact with the Schwann cells’ outer cell membrane (Figure 8; Video S1 in Supplementary Material). One IBA1 cell sent several processes to adjacent dendrite fibers within the osseous spiral lamina. IBA1 cell branches also penetrated into the axon, suggesting that the macrophage process lay inside the myelin layer (Figure 8B, insets). Serial photos and 3D reconstructions verified these conditions. It could not be determined whether IBA1 branches reached the axonal cell membrane at Ranvier nodes or intercellular clefts. IBA1 protein was diffusely expressed within the cytoplasm, and also localized in the cell nuclei (Figure 8, right inset). In several cells, IBA1 protein was associated with the nuclei pores. At higher magnification, irregular patches of staining (100–150 nm) representing cross-sectioned IBA1 branches were seen (Figure 9, inset left). These were submerged within the BL of the Schwann cells. These branches were revealed using SIM but not detectable at confocal microscopy. A few sections contained the VG and neurons. These structures also contained a large number of IBA1-positive cells.

Many IBA1 cells accumulated around the nerve fibers at the habenula perforata where the nerves had lost compact myelin (Figure 7A). These cells also displayed long cytoplasmic processes. No cells or processes invaded the habenula at this point or reached inside the OC. Some of these cells reached into the TCL. These cells often firmly attached to the undersurface of the BM (Figure 7B, inset).

### Fractalkine (CX3CL1) Expression

Fractalkine staining showed only a moderate surface expression of cells within the human OC (Figures 7C,D). No particular difference in staining was noted between hair cells and supporting cells. Some staining was also seen in the cells of the TCL but not at the inferior surface of the BM. Spiral ganglion cells more firmly expressed fractalkine with spotty membrane enhancement also including the nerve fibers (Figures 7E,F).

## Discussion

Super-resolution structured illumination microscopy (SR-SIM), using macrophage immunostaining for marker IBA1, exposed more clearly their interface with neighboring cells. Freshly fixed surgical specimens minimized artifacts that otherwise can influence the microglial phenotype in postmortem material (41). A conceivable weakness of our study was the age of the patients (approximately 40–60 years), as microglia of the aged brain can show an activated immune state (42). Another difficulty is the presence of benign tumors potentially invading the auditory nerve trunk in the respective patients. Nonetheless, such infiltration could not be detected. Subsequently, we consider the samples as representative for steady-state conditions, which is also supported by earlier studies (9).

**Figure 10:**
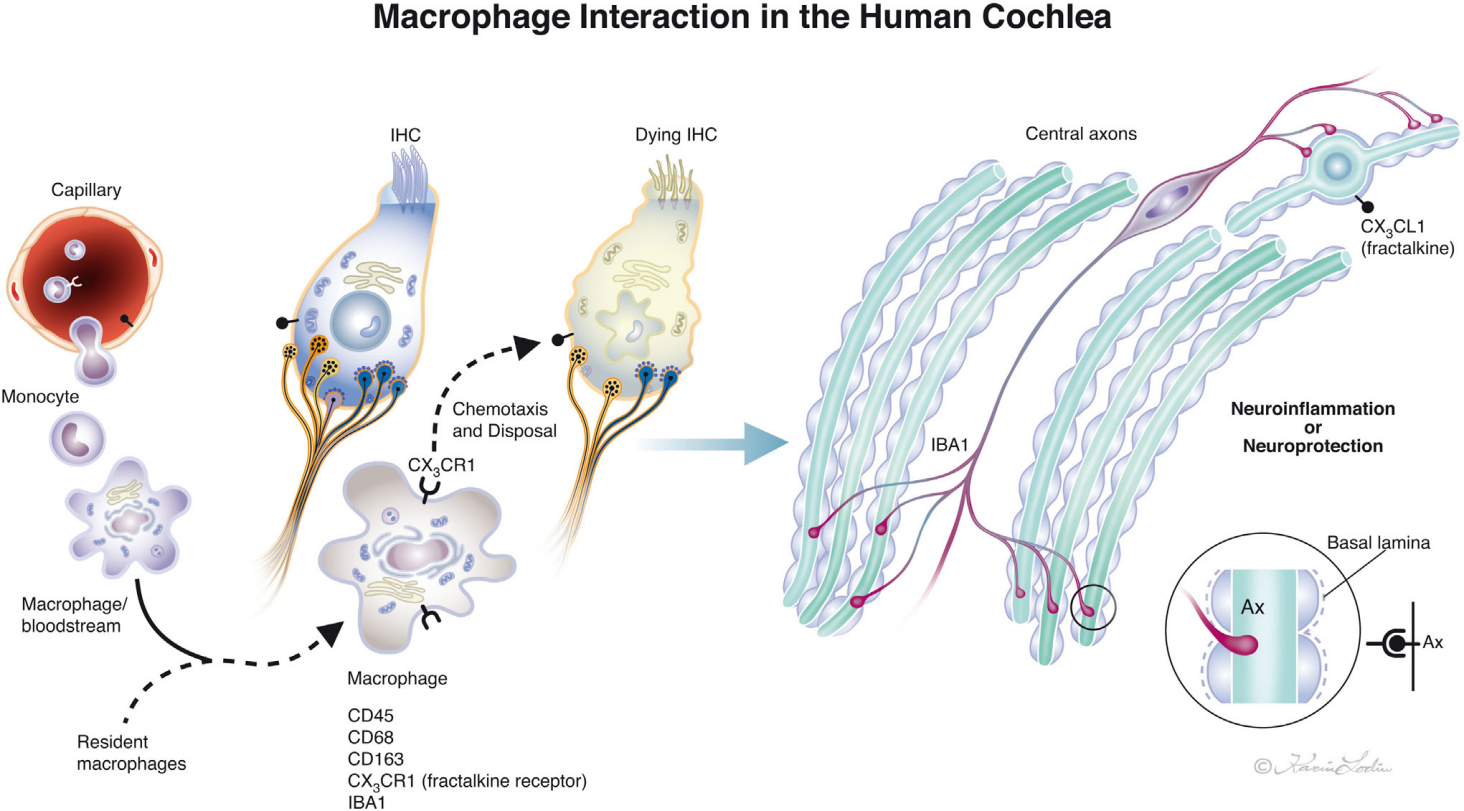
Drawing showing possible macrophage interaction in the human cochlea. Macrophages [ionized calcium-binding adaptor molecule 1 (IBA1) cells] can be observed in the human cochlea, both in the spiral ganglion and more seldom in the organ of corti. Macrophages may interact to form a protective link between hair cells and neurons *via* a fractalkine/CX3CR1 signaling system as demonstrated experimentally by Kaur et al. (23) (illustration by Karin Lodin).

Our study verified that both active and resident IBA1-positive macrophages are widely spread within the human cochlea, including central and peripheral nerve processes. In addition, the OC, which is believed to normally lack immune cell activity (6, 43), contained active macrophages, that could be verified in semi-thin sections as well as in immune stainings. The most impressive localization of IBA1 cells were in the lateral wall and auditory nerve. Unlike resident microglia in the brain, the cochlear macrophages appear to be monocyte derived (7), with turnover rates similar to resident macrophages found in other tissues such as the retina. This result is supported by their presence/transformation inside several stria vessels, presumably prior to diapedesis. The permeability of the strial vessels is otherwise believed to be different to other inner ear vessels (11). The perivascular location suggests a piloting role related to the blood–labyrinth barrier as suggested by Zhang et al. (31) and Shi (30). The highly variable morphology may reflect a high degree of plasticity and adaptation to various microenvironments in the cochlea. In the normal human brain, IBA1-immunoreactive cells display various morphology depending on their stage of activation (ramified, primed, reactive, and amoeboid). In the lateral wall and limbus, they were ramified with round nuclei similar in appearance to primed microglia in the brain (41). The meatal auditory nerve could only be evaluated to a limited degree in these specimens. In the modiolar auditory nerve, they were elongated and had filamentous processes with terminal podosomes attaching adjacent cells. A similar amoeboid pattern was described in the human brain white matter by Torres-Platas et al. (41), where oblong IBA1-IR cells were aligned with myelinated tracts. The distribution of IBA1-positive cells suggests that each nerve fiber may be connected to a ramified macrophage. A linked IBA1 network may survey the environment and protect spiral ganglion neurons. Kaur et al. (23) found that selective loss of cochlear hair cells increased the number of macrophages in the auditory nerve and spiral ligament. These results indicate that there is a link between hair cells and neurons that may influence the auditory nerve preservation under various conditions (Figure 10). Brain studies have shown that, after injury, microglia and infiltrating macrophages can be either pro-inflammatory (M1-like) or immunosuppressive (M2-like). Activated macrophages can release cytotoxic mediators, leading to neuronal dysfunction and cell death. Conversely, they can phagocytose cell debris and secrete neurotrophic factors and anti-inflammatory cytokines to restore tissue integrity (44–46). Thus, macrophages can switch to an anti-inflammatory phenotype that promotes cell differentiation and regeneration (47). Surprisingly, we found that IBA1 branches penetrated and reached the axon surface inside the myelin layer near the axon fiber membrane (Figure 8B, insets). This result was verified through serial sections and 3-D reconstruction (Videos S1 and S2 in Supplementary Material). The entrance point may be the Ranvier node, which further supports the notion that IBA1 macrophages establish direct contact with the axon fiber and even the ganglion cell soma.

Vascular endothelia and several inner ear cells can secrete chemokines that are small signaling cytokines (11). The CX3C chemokine ligand 1, also named fractalkine, induces chemotactic attraction and cell adhesion of macrophages through both membrane-bound and the free form of fractalkine. Increased levels and receptor binding can be seen in different tissues and in various organ-specific human diseases, making it a potential therapeutic target (48–50). The chemokine receptor CX3CR1 is expressed by hematopoietic cells such as specified macrophages, monocytes, natural killer cells, and Th1 lymphocytes (51). In the brain, nerves can be rescued by a modulation of microglia-induced activity by binding to the receptor CX3CR1 and it can even promote adult neurogenesis (52). CX3CR1 reduces inflammatory mediators and leads to secretion of neuroprotective substances such as BDNF (53). In the brain, CX3CL1/CX3CR1 interaction stimulates cytokine secretion from microglia and decrease inflammatory mediators. The neurotrophic effect of CX3CL1 may diminish neuronal death caused by excitotoxicity; an effect depending on extracellular adenosine released from microglia. Our findings of spiral ganglion cells expressing fractalkine including the nerve fibers seem to suggest that macrophage/neuron signaling exists and plays a role in the human cochlear nerve protection. Avoidance of adverse triggering of signaling in connection with cochlear disease and knowledge to exploit their beneficiary properties may lead to new therapeutic possibilities.

High resolution microscopy disclosed remarkable IBA1 cells closely associated with the spiral ganglion cells with “synapse-like” specializations. This may suggest that the human auditory nerve is under the protection and neurotrophic stimulation of a resident macrophage system. It may act across non-myelinated borders of nerve soma and explain why, contrary to most mammals, the human’s auditory nerve is conserved following loss of hair cells and even dendrites. This makes CI possible in man. A representation of the possible interactions between macrophages in the human cochlea is shown in Figure 10. The insertion of prostheses into the cochlea inflicts potential trauma to cochlear tissue (54, 55) that could result in secondary immune responses and activate macrophage signaling. O’Malley et al. (56) presented evidence of foreign body responses in the cochlea to both platinum and silicone after CI, which may be responsible for device failure (57) and electrode extrusion (58). We found evidence that some cells in the TCL are IBA1-positive macrophages. These cells form attachments to the inferior surface of the BM (Figure 7B) which are directly exposed to the electrode array. Therefore, these cells can probably initiate immune reactions at CI. More information about hostile macrophages associated with priming diverse stress against the inner ear and how it can be therapeutically evaded is needed. In the brain, activated microglia signaling can lead to neuroinflammation that is efficiently suppressed by corticosteroids (59) but alternative strategies such as chemokine signaling should also be explored.

In semi-thin sections, migratory macrophages were seen near damaged cochlear hair cells. The cells may clear debris to maintain cellular function and stimulate repair *via* supporting cells to close the spaces after hair cell loss. Furthermore, active IBA1-positive cells were also found to be closely associated with outer hair cells. Whether macrophages are derived from activated resident cells or represent externally recruited cells remains unknown. Findings may corroborate that macrophage-mediated phagocytosis of dead hair cells exists in humans. Macrophage engulfment of vestibular hair cells has earlier been verified, whereas cochlear phagocytosis has been more difficult to verify *in vivo* (23). In time-sequence studies following acoustic overstimulation, active macrophages could be observed within the re-forming sensory epithelium (13). This result suggests that macrophages are actively involved in tissue regeneration. Bone marrow-derived cells, chiefly hematopoietic stem cells negative for the macrophage marker CD45, were found to continuously populate the cochlea and the lateral wall in the adult inner ear (60). Evidence was presented that damaged fibrocytes may be regenerated after injury caused by noise and aminoglycoside exposure. Later, these authors showed that hematopoietic cells can home in and differentiate into macrophages in the adult auditory nerve, i.e., cells that may be used to promote nerve repair or regeneration in the adult inner ear (61). Macrophages may be potential inductors of hair cell proliferation or differentiation, as shown in the avian ear (5, 8, 16, 62) and in fish (25). Hirose et al. (6) suggested that macrophages may enhance tympanic border cells in mice, which could act as latent precursors for regenerated hair cells. If the IBA1 cells associated with OHCs in the present investigation are involved in regeneration, synaptic pruning or degradation activity cannot be established. Even though the mature cochlear epithelium has little regenerative capacity, these and earlier findings of ectopic inner hair cells may indicate that various renewal processes of sensory structures exist in the adult human ear (63). The M2 phenotype microglia-derived cytokines have been found to stimulate proliferation and neuronal differentiation of endogenous stem cells in an ischemic brain (64) and to promote *in vitro* neurogenesis and oligodendrogenesis from nerve stem/progenitor cells (NSPCs) by activating the PPARγ signaling pathway (65). Activation of NSPC differentiation by IL-4 was reported to promote neurogenesis (66). *In vitro* studies have shown that even adult human spiral ganglion cells may have the potential to regenerate, but the cellular mechanisms and whether macrophages play a role in this renewal process remain unknown (67).

Current approaches to renew sensorineural elements in the ear using stem cells may be restricted by endogenous immune responses. To this end, the blood–labyrinth barrier, an equivalent to the blood–brain barrier, may play a crucial role. The blood–labyrinth barrier consists of junctions separating the endolymph from the extracellular tissue. The spiral ganglion is closely related to perilymph and contains fenestrated capillaries. In the adult healthy brain, bone marrow-derived macrophages are restricted to regions lacking a blood–brain barrier. Experiments with mesenchymal stem-cell implantation in the CNS suggest that resident macrophages play a lesser role in the IBA1 inflammatory cell response after cell grafting. Instead, IBA1 cells of monocyte/macrophage origin expressing major histocompatibility complex class II seem to mount a response to grafted cells, even though brain-resident microglia may also be involved (68). Interestingly, resident and infiltrating macrophages may play different roles in neuroinflammation under pathologic conditions, as seen in experimental models of Alzheimer’s disease and MS. The grafting of stem cells in the CNS indicates that the inflammatory response is independent of the fractalkine signaling. It can therefore be expected that stem cell therapy directed to the inner ear may depend on the principal distribution of cells and their relationship to the blood–labyrinth barrier.

## Ethics Statement

The study of human materials was approved by the local ethics committee (Etikprövningsnämnden Uppsala, no. 99398, 22/9 1999, cont, 2003, no. C254/4; no. C45/7 2007, Dnr. 2013/190), and patient consent was obtained. The study adhered to the rules of the Declaration of Helsinki. Archival sections from adult cochleae were used (34, 35). Guinea pig cochleae and brain were analyzed in parallel as controls. Ethical consent was obtained from the local ethical committee of Uppsala for animal use. The guinea pig study’s protocol was approved by the Regional Animal Review Board of Uppsala, Sweden and guinea pig C98/12 and C66/16.

## Author Contributions

WL performed all immunohistochemistry and processing of the human tissue such as fixation, decalcification, and embedding/cryosectioning. He also did confocal and SIM microscopy together with HR-A. He was also instrumental at writing and designing photos. HR-A designed and supervised the whole research project, participated in the research procedures especially interpretation of the research results, photography under both super-resolution SIM and confocal microscopy. HR-A is the main writer of the whole manuscript, and he edited most of the figures. MM was instrumental in performing super-resolution microscopy with all technical issues involved. CG and HB helped to design the project and were instrumental for theory discussions related to inner ear stress induced by cochlear implants to treat the deaf.

## Conflict of Interest Statement

Medel Inc. played a role for the design of the study in connection with cochlear implantation. CG and HB are employed by the Austrian company and are coauthors in this study. WL had 50% salary paid by the Medel company in Austria after contract between the Uppsala University and the Medel Company.

## Supplementary Material

Video S1 3-D reconstruction of TUJ1 (red) and ionized calcium-binding adaptor molecule 1 (green) protein expression in the human spiral ganglion. Both signals were reconstructed with surface rendering mode using the Imaris 8.2 software.

Video S2 3-D reconstruction of collagen IV (red) and ionized calcium-binding adaptor molecule 1 (green) protein expression among dendrites in the osseous spiral lamina. Both signals were reconstructed with surface rendering mode using the Imaris 8.2 software.

## Abbreviations

BC, basal cell; BM, basilar membrane; E, endolymph; EDTA, ethylene-diamine-tetra-acetic acid; IC, intermediate cell; MC, marginal cell; RM, Reissner’s membrane; SM, scala media; SP, spiral prominence; SR-SIM, super-resolution structured illumination fluorescence microscopy; ST, scala tympani; StV, stria vascularis; SV, scala vestibuli; TEM, transmission electron microscopy; IBA1, ionized calcium-binding adaptor molecule 1.

## References

[1] Rask-Andersen H Stahle J Immunodefence of the inner ear? Lymphocyte macrophage interaction in the endolymphatic sac. Acta Otolaryngol (1980) 89:283–94. 10.3109/00016488009127140 7395499

[2] Rask-Andersen H Danckwardt-Lillieström N Friberg U House W. Lymphocyte-macrophage activity in the human endolymphatic sac. Acta Otolaryngol Suppl (1991) 485:15–7. 10.3109/00016489109128039 1843167

[3] Harris JP Ryan AF Immunobiology of the inner ear. Am J Otolaryngol (1984) 5:418–25. 10.1016/S0196-0709(84)80059-9 6400717

[4] Harris JP Heydt J Keithley EM Chen MC. Immunopathology of the inner ear: an update. Ann N Y Acad Sci (1997) 830:166–78. 10.1111/j.1749-6632.1997.tb51888.x 9616676

[5] Warchol ME. Macrophage activity in organ cultures of the avian cochlea: demonstration of a resident population and recruitment to sites of hair cell lesions. J Neurobiol (1997) 33:724–34. 10.1002/(SICI)1097-4695(19971120)33:6<724::AID-NEU2>3.0.CO;2-B 9369147

[6] Hirose K Discolo CM Keasler JR Ransohoff R. Mononuclear phagocytes migrate into the murine cochlea after acoustic trauma. J Comp Neurol (2005) 489:180–94. 10.1002/cne.20619 15983998

[7] Okano T Nakagawa T Kita T Kada S Yoshimoto M Nakahata T Bone marrow-derived cells expressing Iba1 are constitutively present as resident tissue macrophages in the mouse cochlea. J Neurosci Res (2008) 86:1758–67. 10.1002/jnr.21625 18253944

[8] Warchol ME Schwendener RA Hirose K. Depletion of resident macrophages does not alter sensory regeneration in the avian cochlea. PLoS One (2012) 7:e51574. 10.1371/journal.pone.0051574 23240046 PMC3519890

[9] O’Malley JT Nadol JB Jr McKenna MJ. Anti CD163+, Iba1+, and CD68+ cells in the adult human inner ear: normal distribution of an unappreciated class of macrophages/microglia and implications for inflammatory otopathology in humans. Otol Neurotol (2016) 37:99–108. 10.1097/MAO.0000000000000879 26485593 PMC4675683

[10] Steinman RM Cohn ZA Identification of a novel cell type in peripheral lymphoid organs of mice. J Exp Med (1973) 137:1142–62. 10.1084/jem.137.5.1142 4573839 PMC2139237

[11] Hirose K Rutherford MA Warchol ME. Two cell populations participate in clearance of damaged hair cells from the sensory epithelia of the inner ear. Hear Res (2017) 352:70–81. 10.1016/j.heares.2017.04.006 28526177 PMC5544544

[12] Prinz M Erny D Hagemeyer N. Ontogeny and homeostasis of CNS myeloid cells. Nat Immunol (2017) 18:385–92. 10.1038/ni.3703 28323268

[13] Fredelius L. Time sequence of degeneration pattern of the organ of corti after acoustic overstimulation. A transmission electron microscopy study. Acta Otolaryngol (1988) 106:373–85. 10.3109/00016488809107374 3207005

[14] Fredelius L Rask-Andersen H. The role of macrophages in the disposal of degeneration products within the organ of corti after acoustic overstimulation. Acta Otolaryngol (1990) 109:76–82. 10.3109/00016489009107417 2309562

[15] O’Halloran EK Oesterle EC. Characterization of leukocyte subtypes in chicken inner ear sensory epithelia. J Comp Neurol (2004) 475:340–60. 10.1002/cne.20162 15221950

[16] Bhave SA Oesterle EC Coltrera MD Macrophage and microglia-like cells in the avian inner ear. J Comp Neurol (1998) 398:241–56. 10.1002/(SICI)1096-9861(19980824)398:2<241::AID-CNE6>3.0.CO;2-0 9700569

[17] Tornabene SV Sato K Pham L Billings P Keithley EM. Immune cell recruitment following acoustic trauma. Hear Res (2006) 222:115–24. 10.1016/j.heares.2006.09.004 17081714

[18] Ladrech S Wang J Simonneau L Puel JL Lenoir M. Macrophage contribution to the response of the rat organ of corti to amikacin. J Neurosci Res (2007) 85:1970–9. 10.1002/jnr.21335 17497672

[19] Sato E Shick HE Ransohoff RM Hirose K. Repopulation of cochlear macrophages in murine hematopoietic progenitor cell chimeras: the role of CX3CR1. J Comp Neurol (2008) 506:930–42. 10.1002/cne.21583 18085589

[20] Sasaki Y Ohsawa K Kanazawa H Kohsaka S Imai Y Iba1 is an actin-crosslinking protein in macrophages/microglia. Biochem Biophys Res Commun (2001) 286:292–7. 10.1006/bbrc.2001.5388 11500035

[21] Hirose K Sato E Comparative analysis of combination kanamycin furosemide versus kanamycin alone in the mouse cochlea. Hear Res (2011) 272:108e116 10.1016/j.heares.2010.10.011 21044672 PMC4519356

[22] Hirose K Li SZ Ohlemiller KK Ransohoff RM. Systemic lipopolysaccharide induces cochlear inflammation and exacerbates the synergistic ototoxicity of kanamycin and furosemide. J Assoc Res Otolaryngol (2014) 15:555–70. 10.1007/s10162-014-0458-8 24845404 PMC4141430

[23] Kaur T Zamani D Tong L Rubel EW Ohlemiller KK Hirose K Fractalkine signaling regulates macrophage recruitment into the cochlea and promotes the survival of spiral ganglion neurons after selective hair cell lesion. J Neurosci (2015) 35:15050–61. 10.1523/JNEUROSCI.2325-15.2015 26558776 PMC4642237

[24] Yang W Vethanayagam RR Dong Y Cai Q Hu BH. Activation of the antigen presentation function of mononuclear phagocyte populations associated with the basilar membrane of the cochlea after acoustic overstimulation. Neuroscience (2015) 303:1–15. 10.1016/j.neuroscience.2015.05.081 26102003 PMC4532582

[25] Carrillo SA Anguita-Salinas C Pena OA Morales RA Munoz-Sanchez S Munoz-Montecinos C Macrophage recruitment contributes to regeneration of mechanosensory hair cells in the zebrafish lateral line. J Cell Biochem (2016) 117:1880–9. 10.1002/jcb.25487 26755079

[26] Li L Nevill G Forge A. Two modes of hair cell loss from the vestibular sensory epithelia of the guinea pig inner ear. J Comp Neurol (1995) 355:405–17. 10.1002/cne.903550307 7636022

[27] Abrashkin KA Izumikawa M Miyazawa T Wang CH Crumling MA Swiderski DL The fate of outer hair cells after acoustic or ototoxic insults. Hear Res (2006) 218:20–9. 10.1016/j.heares.2006.04.001 16777363

[28] Anttonen T Belevich I Kirjavainen A Laos M Brakebusch C Jokitalo E How to bury the dead: elimination of apoptotic hair cells from the hearing organ of the mouse. J Assoc Res Otolaryngol (2014) 15:975–92. 10.1007/s10162-014-0480-x 25074370 PMC4389953

[29] Monzack EL May LA Roy S Gale JE Cunningham LL. Live imaging the phagocytic activity of inner ear supporting cells in response to hair cell death. Cell Death Differ (2015) 22:1995–2005. 10.1038/cdd.2015.48 25929858 PMC4816108

[30] Shi X. Resident macrophages in the cochlear blood-labyrinth barrier and their renewal via migration of bone-marrow-derived cells. Cell Tissue Res (2010) 342:21–30. 10.1007/s00441-010-1040-2 20838812

[31] Zhang W Dai M Fridberger A Hassan A Degagne J Neng L Perivascular-resident macrophage-like melanocytes in the inner ear are essential for the integrity of the intrastrial fluid-blood barrier. Proc Natl Acad Sci U S A (2012) 109:10388–93. 10.1073/pnas.1205210109 22689949 PMC3387119

[32] Ohsawa K Imai Y Kanazawa H Sasaki Y Kohsaka S. Involvement of Iba1 in membrane ruffling and phagocytosis of macrophages/microglia. J Cell Sci (2000) 113:3073–84. 10934045 10.1242/jcs.113.17.3073

[33] Frick LR Williams K Pittenger C. Microglial dysregulation in psychiatric disease. Clin Dev Immunol (2013) 2013:608654. 10.1155/2013/608654 23690824 PMC3652125

[34] Liu W Li H Edin F Brännström J Glueckert R Schrott-Fischer A Molecular composition and distribution of gap junctions in the sensory epithelium of the human cochlea-a super-resolution structured illumination microscopy (SR-SIM) study. Ups J Med Sci (2017) 17:1–11. 10.1080/03009734.2017.1322645 28513246 PMC5649321

[35] Liu W Schrott-Fischer A Glueckert R Benav H Rask-Andersen H The human “Cochlear Battery”. Claudin-11 barrier and ion transport proteins in the lateral wall of the cochlea. Front Mol Neurosci (2017) 10:239 10.3389/fnmol.2017.00239 28848383 PMC5554435

[36] Lee-Kubli CA Ingves M Henry KW Shiao R Collyer E Tuszynski MH Analysis of the behavioral, cellular and molecular characteristics of pain in severe rodent spinal cord injury. Exp Neurol (2016) 278:91–104. 10.1016/j.expneurol.2016.01.009 26808661 PMC13189222

[37] Truman LA Ford CA Pasikowska M Pound JD Wilkinson SJ Dumitriu IE CX3CL1/fractalkine is released from apoptotic lymphocytes to stimulate macrophage chemotaxis. Blood (2008) 112:5026–36. 10.1182/blood-2008-06-162404 18799722

[38] Gustafsson MG Shao L Carlton PM Wang CJ Golubovskaya IN Cande WZ Three-dimensional resolution doubling in wide-field fluorescence microscopy by structured illumination. Biophys J (2008) 94:4957–70. 10.1529/biophysj.107.120345 18326650 PMC2397368

[39] Tylstedt S Rask-Andersen H A 3-D model of membrane specializations between human auditory spiral ganglion cells. J Neurocytol (2001) 30:465–73. 10.1023/A:1015628831641 12037463

[40] Pamulova L Linder B Rask-Andersen H. Innervation of the apical turn of the human cochlea: a light microscopic and transmission electron microscopic investigation. Otol Neurotol (2006) 27:270–5. 10.1097/01.mao.0000187239.56583.d2 16437000

[41] Torres-Platas SG Comeau S Rachalski A Bo GD Cruceanu C Turecki G Morphometric characterization of microglial phenotypes in human cerebral cortex. J Neuroinflammation (2014) 11:11–2. 10.1186/1742-2094-11-12 24447857 PMC3906907

[42] Norden DM Godbout JP. Review: microglia of the aged brain: primed to be activated and resistant to regulation. Neuropathol Appl Neurobiol (2013) 39:19–34. 10.1111/j.1365-2990.2012.01306.x 23039106 PMC3553257

[43] Du X Choi CH Chen K Cheng W Floyd RA Kopke RD. Reduced formation of oxidative stress biomarkers and migration of mononuclear phagocytes in the cochleae of chinchilla after antioxidant treatment in acute acoustic trauma. Int J Otolaryngol (2011) 2011:612690. 10.1155/2011/612690 21961007 PMC3179894

[44] David S Kroner A. Repertoire of microglial and macrophage responses after spinal cord injury. Nat Rev Neurosci (2011) 12:388–99. 10.1038/nrn3053 21673720

[45] Kumar A Loane DJ. Neuroinflammation after traumatic brain injury: opportunities for therapeutic intervention. Brain Behav Immun (2012) 26:1191–201. 10.1016/j.bbi.2012.06.008 22728326

[46] Loane DJ Kumar A. Microglia in the TBI brain: the good, the bad, and the dysregulated. Exp Neurol (2016) 275:316–27. 10.1016/j.expneurol.2015.08.018 26342753 PMC4689601

[47] Saclier M Yacoub-Youssef H Mackey AL Arnold L Ardjoune H Magnan M Differentially activated macrophages orchestrate myogenic precursor cell fate during human skeletal muscle regeneration. Stem Cells (2013) 31:384–96. 10.1002/stem.1288 23169615

[48] Fuhrmann M Bittner T Jung CKE Burgold S Page RM Mitteregger G Microglial Cx3cr1 knockout prevents neuron loss in a mouse model of Alzheimer’s disease. Nat Neurosci (2010) 13:411–3. 10.1038/nn.2511 20305648 PMC4072212

[49] Nanki T Imai T Kawai S. Fractalkine/CX3CL1 in rheumatoid arthritis. Mod Rheumatol (2017) 27:392–7. 10.1080/14397595.2016.1213481 27484962

[50] Erreni M Siddiqui I Marelli G Grizzi F Bianchi P Morone D The fractalkine-receptor axis improves human colorectal cancer prognosis by limiting tumor metastatic dissemination. J Immunol (2016) 196:902–14. 10.4049/jimmunol.1501335 26673138

[51] Imai T Hieshima K Haskell C Baba M Nagira M Nishimura M Identification and molecular characterization of fractalkine receptor CX3CR1, which mediates both leukocyte migration and adhesion. Cell (1997) 91:521–30. 10.1016/S0092-8674(00)80438-9 9390561

[52] Sellner S Paricio-Montesinos R Spieß A Masuch A Erny D Harsan LA Microglial CX3CR1 promotes adult neurogenesis by inhibiting Sirt 1/p65 signaling independent of CX3CL1. Acta Neuropathol Commun (2016) 4:102. 10.1186/s40478-016-0374-8 27639555 PMC5027111

[53] Lauro C Cipriani R Catalano M Trettel F Chece G Brusadin V Adenosine A1 receptors and microglial cells mediate CX3CL1-induced protection of hippocampal neurons against Glu-induced death. Neuropsychopharmacology (2010) 35:1550–9. 10.1038/npp.2010.26 20200508 PMC3055460

[54] Kamakura T Nadol JB Jr. Cochlear histopathology as observed in two patients with a cochlear implant electrode with positioner. Otol Neurotol (2016) 37:642–6. 10.1097/MAO.0000000000000976 27273406 PMC4907849

[55] Quesnel AM Nakajima HH Rosowski JJ Hansen MR Gantz BJ Nadol JB Jr. Delayed loss of hearing after hearing preservation cochlear implantation: human temporal bone pathology and implications for etiology. Hear Res (2016) 333:225–34. 10.1016/j.heares.2015.08.018 26341474 PMC4775460

[56] O’Malley JT Burgess BJ Galler D Nadol JB Jr. Foreign body response to silicone in cochlear implant electrodes in the human. Otol Neurotol (2017) 8:970–7. 10.1097/MAO.0000000000001454 28538471 PMC5500409

[57] Nadol JB Jr Eddington DK Burgess BJ. Foreign body or hypersensitivity granuloma of the inner ear after cochlear implantation: one possible cause of a soft failure? Otol Neurotol (2008) 29:1076–84. 10.1097/MAO.0b013e31818c33cf 18997635 PMC4180287

[58] Benatti A Castiglione A Trevisi P Bovo R Rosignoli M Manara R Endocochlear inflammation in cochlear implant users: case report and literature review. Int J Pediatr Otorhinolaryngol (2013) 77:885–93. 10.1016/j.ijporl.2013.03.016 23578804

[59] Sugama S Takenouchi T Fujita M Kitani H Conti B Hashimoto M. Corticosteroids limit microglial activation occurring during acute stress. Neuroscience (2013) 232:13–20. 10.1016/j.neuroscience.2012.12.012 23262242

[60] Lang H Ebihara Y Schmiedt RA Minamiguchi H Zhou D Smythe N Contribution of bone marrow hematopoietic stem cells to adult mouse inner ear: mesenchymal cells and fibrocytes. J Comp Neurol (2006) 496:187–201. 10.1002/cne.20929 16538683 PMC2561311

[61] Lang H Nishimoto E Xing Y Brown LN Noble KV Barth JL Contributions of mouse and human hematopoietic cells to remodeling of the adult auditory nerve after neuron loss. Mol Ther (2016) 24:2000–11. 10.1038/mt.2016.174 27600399 PMC5154482

[62] Warchol ME. Immune cytokines and dexamethasone influence sensory regeneration in the avian vestibular periphery. J Neurocytol (1999) 28:889–900. 10.1023/A:1007026306730 10900092

[63] Rask-Andersen H Li H Löwenheim H Müller M Pfaller K Schrott-Fischer A Supernumerary human hair cells-signs of regeneration or impaired development? A field emission scanning electron microscopy study. Ups J Med Sci (2017) 122:11–9. 10.1080/03009734.2016.1271843 28145795 PMC5361427

[64] Choi JY Kim JY Kim JY Park J Lee WT Lee JE. M2 phenotype microglia-derived cytokine stimulates proliferation and neuronal differentiation of endogenous stem cells in ischemic brain. Exp Neurobiol (2017) 26:33–41. 10.5607/en.2017.26.1.33 28243165 PMC5326713

[65] Yuan J Ge H Liu W Zhu H Chen Y Zhang X M2 microglia promotes neurogenesis and oligodendrogenesis from neural stem/progenitor cells via the PPARγ signaling pathway. Oncotarget (2017) 8:19855–65. 10.18632/oncotarget.15774 28423639 PMC5386728

[66] Butovsky O Ziv Y Schwartz A Landa G Talpalar AE Pluchino S Microglia activated by IL-4 or IFN-gamma differentially induce neurogenesis and oligodendrogenesis from adult stem/progenitor cells. Mol Cell Neurosci (2006) 31:149–60. 10.1016/j.mcn.2005.10.006 16297637

[67] Rask-Andersen H Boström M Gerdin B Kinnefors A Nyberg G Engstrand T Regeneration of human auditory nerve. In vitro/in video demonstration of neural progenitor cells in adult human and guinea pig spiral ganglion. Hear Res (2005) 203:180–91. 10.1016/j.heares.2004.12.005 15855043

[68] Le Blon D Hoornaert C Daans J Santermans E Hens N Goossens H Distinct spatial distribution of microglia and macrophages following mesenchymal stem cell implantation in mouse brain. Immunol Cell Biol (2014) 92:650–8. 10.1038/icb.2014.49 24983456

